# Increased Risk of Psoriasis due to combined effect of HLA-Cw6 and LCE3 risk alleles in Indian population

**DOI:** 10.1038/srep24059

**Published:** 2016-04-06

**Authors:** Aditi Chandra, Anirudhya Lahiri, Swapan Senapati, Baidehi Basu, Saurabh Ghosh, Indranil Mukhopadhyay, Akhilesh Behra, Somenath Sarkar, Gobinda Chatterjee, Raghunath Chatterjee

**Affiliations:** 1 Human Genetics Unit, Indian Statistical Institute, 203 B. T. Road, Kolkata, India 700108; 2 Consultant Dermatologist, Uttarpara, Hooghly India 712258; 3 Department of Dermatology, SSKM Hospital, Kolkata, India; 4 Department of Dermatology, School of Tropical medicine, Kolkata, India

## Abstract

HLA-Cw6 is one of the most associated alleles in psoriasis. Recently, Late Cornified Envelop 3 (LCE3) genes were identified as a susceptibility factor for psoriasis. Some population showed epistatic interaction of LCE3 risk variants with HLA-Cw6, while some population failed to show any association. We determined the associations of a 32.2 kb deletion comprising LCE3C-3B genes and three SNPs (rs1886734, rs4112788; rs7516108) at the LCE3 gene cluster among the psoriasis patients in India. All three SNPs at the LCE3 gene cluster failed to show any association. In contrary, for patients with HLA-Cw6 allele, all three SNPs and the LCE3C-3B deletion showed significant associations. While, all five LCE3 genes were upregulated in psoriatic skin, only LCE3A showed significant overexpression with homozygous risk genotype compared to the non-risk genotype. LCE3B also showed significant overexpression in patients with HLA-Cw6 allele. Moreover, LCE3A showed significantly higher expression in patients bearing homozygous risk genotype in presence of HLA-Cw6 allele but not in those having non-risk genotype, demonstrating the combined effect of HLA-Cw6 allele and risk associated genotype near LCE3A gene. Integration of genetic and gene expression data thus allowed us to identify the actual disease variants at the LCE3 cluster among the psoriasis patients in India.

Psoriasis is a recurrent chronic inflammatory skin disorder, caused by hyper-proliferating keratinocytes that fail to differentiate properly, leading to characteristic psoriatic plaque formation^1^. The prevalence of the disease varies form 0.2% to 11.8% across the populations worldwide^1^,^2^,^3^,^4^. In India, 0.44–2.8% of the population are affected by psoriasis^4^. Although the disease is not usually fatal, but it leads to physical discomfort and tremendous psychological stress, which ultimately affects the patients’ professional and social life^5^. Approximately 15–40% of psoriasis patients may develop inflammatory arthritis^6^,^7^.

Psoriasis has been studied as a separate skin disease since early nineteenth century^8^; but the exact mechanism of disease trigger is still largely unknown. Initial genetic studies revealed the most prominent disease marker at chromosome 6p21 (PSORS1)^9^,^10^,^11^,^12^. HLA-Cw6 was identified as the major risk allele within this region^11^. However, the penetrance of this allele was estimated to be only 10%, suggested involvement of other susceptibility loci^13^. Later studies identified association with several other genetic loci (PSORS1–13) among different populations worldwide^14^,^15^,^16^,^17^,^18^,^19^,^20^,^21^,^22^,^23^,^24^. A linkage-based study on three-generation Italian families identified PSORS4 at chromosome 1q21 to be associated with the disease^25^. Finer mapping revealed that the PSORS4 susceptibility locus was included within the Epidermal Differentiation Complex (EDC), a cluster of 27 genes; many of them were expressed during the epidermal differentiation^26^,^27^. Aberrant differentiation and improper maturation of the epithelium is a key pathogenic event in psoriasis^28^. Primary focus, therefore, was on investigating the role of genes in the EDC^29^,^30^,^31^. Furthermore, recent genome-wide association studies (GWASs) on Chinese-Han population demonstrated association of the disease with SNPs within LCE3 (Late Cornified Envelop) gene cluster^18^,^32^,^33^. LCE3 gene cluster spans ~60 kb and comprises five genes (LCE3A, 3B, 3C, 3D and 3E) within the EDC (PSORS4) at chromosome 1q21. Because of the strategic role of these genes in epidermal barrier repair and their reported upregulation in psoriatic skin^34^, many later studies focused on the LCE3 genes^35^,^36^,^37^,^38^,^39^. It has previously been demonstrated that the expression of LCE3 genes were markedly induced after superficial injury of the normal skin^34^,^40^. Stimulation of *in vitro* reconstructed skin with psoriasis-associated cytokines (IL-1α, TNFα, IL-6) also significantly upregulated LCE3 expressions^34^.

Deletion of a 32.2 kb region at the EDC comprising LCE3C and LCE3B genes was also found to be associated with psoriasis^40^. Association of LCE3C-3B copy number variation (CNV) and its epistatic interaction with HLA-Cw6 with the disease were observed in some European and Asiatic^41^, German^42^ and northern Chinese population^33^. In contrast, LCE3C-3B-del was not associated with the psoriasis in Tunisian^43^ and Japanese population^41^, indicated the population specific differences in association of this cluster. Most of the studies among Indian population identified HLA-Cw6 as the most strongly associated loci^15^,^44^,^45^. The association of LCE3 cluster with psoriasis has not been determined among Indian population. Most importantly, the functional implications of the combined effect of these risk variants were also not reconnoitered.

In the present study, we have determined the involvement of all five LCE3 genes in the pathogenesis of psoriasis among Indian patients. We have analyzed the association of the LCE3C-3B deletion and three SNPs (rs1886734, rs4112788, rs7516108) within the LCE3 cluster among psoriasis patients in India. We determined the epistatic interaction of HLA-Cw6 and the LCE3 cluster, and finally appraised the functional implications of the combined effect of HLA-Cw6 and LCE3 risk variants.

## Results

### Association of HLA-Cw6 allele with psoriasis

The presence of HLA-Cw6 allele was determined for 705 psoriasis cases and 738 healthy controls using sequence specific PCR (SSP). The mean age of psoriasis patients, recruited from the eastern part of India, was 40.98 years (SD = 15.8) and that of the disease onset was 34.96 years (SD = 15.68). The mean age of the healthy individuals was 39.24 years (SD = 14.72) (Supplementary Table S1). We observed evidence of significant association of HLA-Cw6 allele with psoriasis (OR = 4.93, 95% CI: 3.83–6.34; P-value < 2.2 × 10^−16^). Among the 705 psoriasis cases, 61.74% had type-I form of the disease (age of onset ≤40 years). Approximately 53% of the type-I cases and 34% of the type-II cases (age of onset >40 years) carried the HLA-Cw6 risk allele, while only 14.85% of normal individuals had this allele (Supplementary Table S2). Unlike previously reported^46^,^47^, we obtained significant association of HLA-Cw6 allele for both type-I and type-II patients in comparison to the controls (type-I: P-value < 2.2 × 10^−16^; and type-II: P-value = 1.38 × 10^−11^). Nonetheless, the risk imparted by HLA-Cw6 was more than two fold in type-I patients (OR = 6.61) compared to type-II patients (OR = 2.98) (Supplementary Table S2).

### Association of LCE3 gene cluster with psoriasis

We genotyped three previously reported SNPs (rs1886734, rs4112788, rs7516108)^18^ within the LCE3 gene cluster (chr1q21) as well as determined the status of a 32.2 kb deletion comprising LCE3B and LCE3C genes^40^. All SNPs and the LCE3C-3B deletion were found to be in Hardy-Weinberg equilibrium in both psoriasis cases and healthy controls (P-value > 0.05) (Supplementary Table S3). In allele level association analysis, we observed significant increase in risk for the SNP (rs7516108) near LCE3E (OR = 1.19, P-value = 0.0269) and the deleted variant of LCE3C-3B (OR = 1.27, P-value = 0.0018) (Table 1). Similar trend for the SNPs near LCE3A (rs1886734) and LCE3D (rs4112788) were observed but they did not reach the level of significance at 0.05 (LCE3A:OR = 1.16, P-value = 0.0578; and LCE3D:OR = 1.16, P-value = 0.0578). This is in contrast to the previously reported high degree of association observed in the Chinese population^18^. The deleted variant of LCE3C-3B genes was overall the more frequent allele among the psoriasis patients in India, and showed significant increase in risk even after multiple testing correction. We evaluated the genotype level association of these SNPs and the LCE3C-3B deletion after adjusting for age and sex. Only the LCE3C-3B deleted allele showed significant increase in risk (Supplementary Table S4), whereas LCE3E (rs7516108) did not reach the level of significance.

We determined the linkage disequilibrium (LD) by estimating the r^2^ for three SNPs and the LCE3C-3B deletion. LCE3 cluster was found to be in strong LD in the eastern Indian population (Supplementary Fig. S1, Supplementary Table S5). SNPs near LCE3A (rs1886734) and LCE3D (rs4112788) showed the strongest linkage (r^2^ = 0.971) within the LCE3 cluster. We also observed the presence of a strong linkage pattern of LCE3C-3B deletion with both SNPs rs4112788 (r^2^ = 0.813) and rs1886734 (r^2^ = 0.806).

The combined effect of HLA-Cw6 allele and the LCE3 gene cluster were estimated using binary logistic regression using HLA-Cw6 as a covariate. LCE3C-3B deletion and all three SNPs at the LCE3 gene cluster did not show any significant association, indicating that the HLA-Cw6 allele might have some role for LCE3 risk alleles to exert their effect. As the frequency of HLA-Cw6 allele is much higher in type-I in comparison to the type-II patients, we wanted to determine if the patients had any different genetic associations for type-I and type-II patients (Supplementary Table S6). Significantly higher association was observed for LCE3C-3B deletion and the SNPs near LCE3A, LCE3D and LCE3E genes for type-I patients, while no significant association of any of the LCE3 genes were observed for type-II patients (Supplementary Table S6). This could be the consequence of higher frequency of HLA-Cw6 positive patients under type-I group, again indicating the combined effect of HLA-Cw6 and LCE3 cluster on psoriasis susceptibility. To further identify if LCE3 genes had any interaction with HLA-Cw6, we classified the patients based on the presence of HLA-Cw6 allele and studied the combined effect of HLA-Cw6 and LCE3 risk alleles.

Both LCE3C-3B deletion and the SNP near LCE3E gene (rs7516108) showed the most significant association under dominant model (P-value = 0.00987 and 0.0041) (Supplementary Table S7). Considering the high linkage pattern within the LCE3 cluster, we assumed all of LCE3 SNPs for further analyses to operate according to the dominant model of association. The non-risk genotype was considered as reference. The risk variants of the total LCE3 cluster conferred significant risk only in presence of HLA-Cw6 allele (Table 2). In contrast, no significant difference in risk was observed in absence of HLA-Cw6 allele (Table 2). Notably, the risk was increased by ~2 fold for all studied loci of HLA-Cw6 positive patients bearing LCE3 risk variants in comparison to the non-risk variants. Epistatic interaction of HLA and LCE3, as reported in some European populations^41^ and Chinese population^48^, is also evident in Indian psoriasis patients. This analysis indicated the possible genetic heterogeneity between HLA-Cw6 positive and negative psoriasis patients. Stratifying the samples based on the presence of HLA-Cw6 showed significantly high association of all studied risk alleles at the LCE3 gene cluster (Table 3). Similar results were obtained after adjusting for age and sex (Supplementary Table S8). This analysis clearly supported our notion of genetic heterogeneity between HLA-Cw6 positive and negative patients, and suggested that they should be considered separately.

### Expressions of LCE3 genes in the psoriatic skin of patients with risk alleles

**Figure 1:**
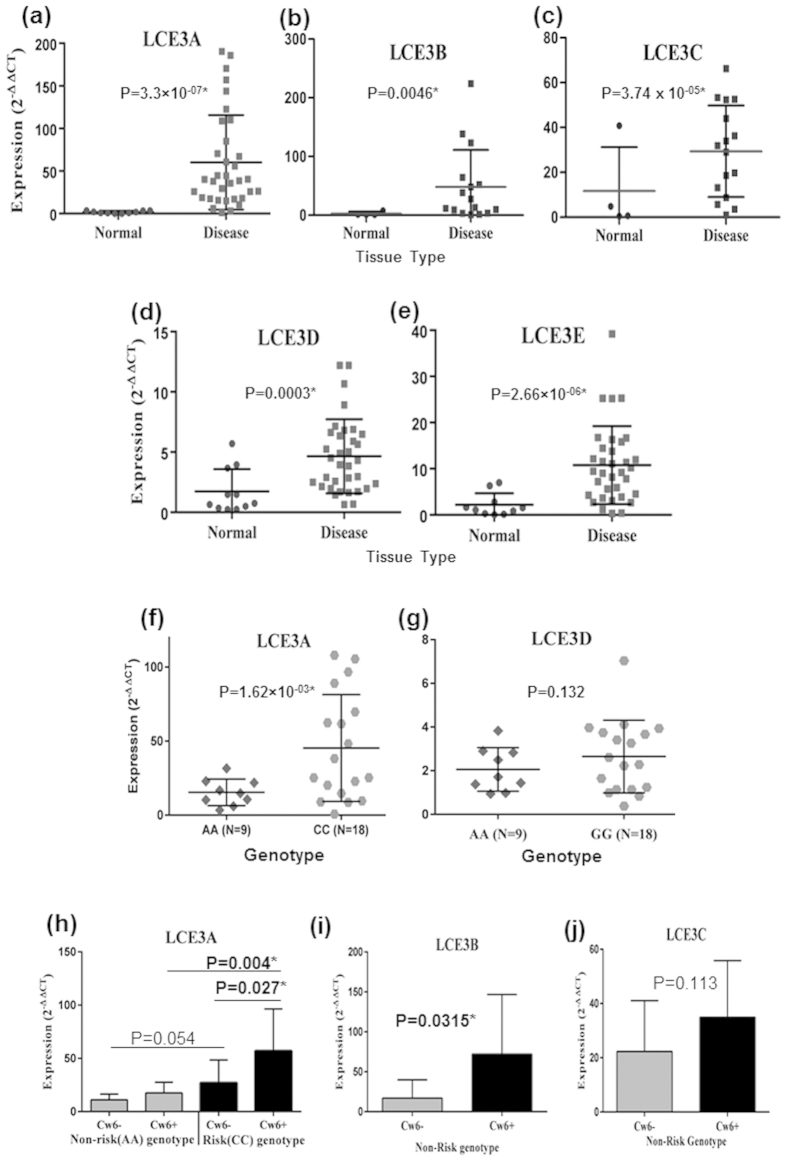
Expression profiling of LCE3 genes. (**a**–**e**) Expression pattern of five LCE3 genes in psoriatic and adjacent normal skin. All five LCE3 genes were significantly upregulated in psoriatic skin. (**f**–**g**) Variation in expression pattern from diseased tissue with respect to genotypes of LCE3 genes. Only LCE3A expression was significantly upregulated with risk genotype. (**h**–**i**) Expression pattern of LCE3 genes with respect to genotype and HLA-Cw6 status. Only LCE3A showed significant difference between homozygous non-risk and risk genotype in presence of HLA-Cw6. Error bars represents the Standard deviation.

We showed the combined genetic association of LCE3 and HLA-C risk alleles in Indian psoriasis patients. SNPs near LCE3 genes and the LCE3C-3B deletion were found to be in strong LD in eastern Indian population, suggested that the association signals from various SNPs in this region were not independent. This could be a consequence of the strong LD with the actual causal variant. Under such conditions, it was difficult to detect the actual causal variant without further functional study. To determine the levels of transcripts encoding LCE3A, 3B, 3C, 3D and 3E in psoriatic skin, we performed quantitative real time PCR (qRT-PCR) on RNA isolated from uninvolved and involved psoriatic skin. We observed significantly increased expression of LCE3A (P-value = 3.3 × 10^−7^), LCE3B (P-value = 0.0046), LCE3C (P-value = 3.74 × 10^−5^), LCE3D (P-value = 3.50 × 10^−4^) and LCE3E (P-value = 2.66 × 10^−6^) mRNA in the involved skin compared to the uninvolved one (Fig. 1a–e). To detect if the genetic interactions had any biological effect in terms of its expression, we examined the expression pattern of all LCE3 genes in the involved skin of patients with LCE3 risk and non-risk genotypes. Only, the LCE3A risk genotype showed significantly higher expression in comparison to the non-risk genotype (P-value = 0.0016) (Fig. 1f,g, Supplementary Fig. S1b). Interestingly, LCE3A gene had significantly higher expression in patients bearing HLA-Cw6 and LCE3A risk allele (P-value = 0.027) in comparison to the patients with LCE3A risk allele but in absence of HLA-Cw6 allele. Furthermore, HLA-Cw6 positive individuals with LCE3A risk allele showed significantly higher expression in comparison to the HLA-Cw6 positive and LCE3A non-risk allele carriers (P-value = 0.004) (Fig. 1h). Similar high expressions of LCE3B and LCE3C genes were also observed in carriers of both LCE3C-3B non-risk allele (homozygous or heterozygous) and HLA-Cw6 allele, but was significant only in case of LCE3B (P-value = 0.0315) (Fig. 1i,j). In contrast, LCE3D and LCE3E did not show any significant changes in expression between risk and non-risk carriers (Fig. 1g, Supplementary Fig. S1b,c). This shows that the genetic association of LCE3 genes and its interaction with HLA-Cw6 allele are also reflected in terms of expression of specific LCE3 genes.

## Discussion

HLA-Cw6 is one of the most commonly reported and significantly associated alleles with psoriasis irrespective of population^11^,^48^. It was found to be associated mainly for the type-I patients with generalized plaque type psoriasis^46^,^47^,^49^. Deletion of LCE3C-3B also showed significant association with the disease in several populations^32^,^40^,^42^. Several reports suggested combined effect of HLA-Cw6 with LCE3 genes^41^,^48^. In contrary, population specific differences are also observed^41^,^43^,^50^. This suggests the importance of examining genetic risk factors in multiple populations. The functional significance of these risk variants near LCE3 genes, to our knowledge, is not determined yet. Moreover, any such association study among the Indian psoriasis patients is also lacking. This is, to our knowledge, the first report among the Indian patient to determine the genetic association of the entire LCE3 gene cluster. We have also shown the functional implications of this genetic association. Furthermore, our study points out that due to the underlying genetic heterogeneity, it could be more appropriate to stratify the patients according to HLA-Cw6 status to elucidate the actual genetic associations.

HLA-Cw6 was significantly associated with the disease. However, SNPs at the LCE3 cluster failed to show any association, although the frequency of risk alleles was higher in disease population. Interestingly, the combined effect of LCE3 and HLA-Cw6 risk alleles increased the risk of disease by two folds. Phenotypically, the presence of the risk allele increased the expression of LCE3A transcripts by more than three times (P-value = 0.0016). We observed highly variable expression pattern of LCE3A gene in the involved skin of psoriasis patients in comparison to the uninvolved skin (Fig. 1a). While these patients were grouped based on the LCE3A risk and non-risk genotypes, a significant difference was observed between these groups (Fig. 1f). Furthermore, when LCE3A risk variants were classified based on the HLA-Cw6 risk allele, the LCE3A gene transcript was significantly upregulated compared to the cases where HLA-Cw6 was absent (Fig. 1h). Our data, thus present a functional implication of the combined effect of HLA-Cw6 and LCE3.

LCE3 risk alleles were present in 64% of the normal individuals in our samples, but the risk of psoriasis was reported to be only 0.44–2.8%. This indicated the involvement of other genetic susceptibility factors along with the LCE3 genes to cause the disease. Our data suggested that the combination of HLA-Cw6 and LCE3 risk alleles might be a susceptibility factor for the psoriasis. The high frequency of early age of disease onset, HLA-Cw6 positive and familial psoriasis patients in Chinese GWAS^18^ could be the reason for high association observed for the LCE3 cluster. However, a smaller sample size in comparison to the Chinese GWAS can not be ruled out for this lack of association in our study. Our samples included both early and late-onset patients. Type-I cases, which were enriched with HLA-Cw6, showed significant association with the LCE3 cluster, while failed to show any association when both type-I and type-II cases were considered together. This again indicated the genetic heterogeneity between HLA-Cw6 positive and negative cases, and suggested that they should be considered separately to identify the actual risk loci.

Several studies have reported significant upregulation of LCE3 transcripts in psoriatic skin compared to healthy/uninvolved skin^34^,^40^. Tape stripping or superficial injuries to the skin also caused similar effects^34^. We have shown here that the presence of risk alleles caused overexpression of specific LCE3 genes. However, this is in sharp contrast to the fact that analogs of Vitamin D3 used to treat psoriasis (1,25-dihydroxyvitamin D3) functions by increasing LCE3 expression, which subsequently heals the disease^35^. Higher LCE3 expression thus appears as protective. To solve this riddle, we hypothesize that the actual disease-causing variant within LCE3 cluster may be the deleted LCE3C-3B genes, which are in strong LD with other LCE3 risk alleles. Strong association signals from the other LCE3 loci are not independent, but are the consequence of strong linkage disequilibrium in this region. LCE3C-3B deletion is observed in more than 90% of the patients with LCE3A and LCE3D risk allele. LCE3A gene expression is also significantly upregulated in risk allele carriers in comparison to the non-risk ones. Considering these observations, we can speculate that in psoriasis patients with LCE3C-3B deletion, LCE3A gene is upregulated to compensate the barrier repair function of LCE3C-3B genes. Upregulation of LCE3A might be an effect, not the triggering cause of the disease. Vitamin D3 analogs or plant-anthocyanidins can heal psoriasis only in those patients who may have atleast one intact copy of LCE3C-3B. The 40% non-responders^51^ could be the patients who entirely lack LCE3C-3B genes. However, extensive validations are required to test the generality of our hypothesis among different populations worldwide.

## Materials and Methods

### Study Population

Psoriasis cases (N = 705) and healthy controls (N = 738) were recruited from multiple hospitals in the eastern region of India. The clinical diagnoses of psoriasis patients were confirmed by at least two dermatologists. All healthy controls were clinically assessed as being without psoriasis, other autoimmune disorders, systemic disorders or without a family history of psoriasis in the first and second degree relatives. Patients and controls gave their informed consent; for children younger than 18 years of age, consent was also obtained from their parents. The study was approved by the Institutional Ethics Committee for Human Research, Indian Statistical Institute, Kolkata, India and conducted according to the Declaration of Helsinki Principles. Only patients categorized as having plaque or guttate psoriasis were enrolled in the study to minimize clinical heterogeneity. Three ml of blood sample was collected from each patient. Uninvolved and involved skin biopsies (4 mm) were obtained from 36 psoriasis patients. Biopsy specimens were collected in RNA Later (Invitrogen) and stored at −80 °C until processing.

### HLA-Cw6 allele typing

Genomic DNA was isolated using Qiagen Blood/Tissue DNA isolation kit according to the manufacturer’s protocol. HLA-Cw6 typing was carried out using sequence specific PCR (SSP) as described previously^52^ and referred to as positive when the HLA-Cw6 allele was present and negative when not present. The sequence specific primers were (HLA-Cw6_F: 5′-TACTACAACCAGAGCGAGGA-3′; HLA-Cw6_R: 5′-GGTCGCAGCCATACATCCA-3′). Positive internal control amplicon with primer sequences (F: 5′-TGCCAAGTGGAGCACCCAA-3′; R: 5′-GCATCTTGCTCTGTGCAGAT-3′) giving a 779 bps product (HLA-DRB1 intron sequence) was run with each sample to check for PCR failure.

### SNP selection and Genotyping

Three SNPs were selected for genotyping that map to the 1q21 locus comprising Epidermal Differentiation Complex (EDC) (LCE3A: rs1886734, LCE3D: rs4112788; LCE3E: rs7516108). Two SNPs, rs1886734 and rs4112788, have previously shown to be significantly associated in Chinese population^18^. Two independent studies reported rs4112788 to be the most associated variant^18^,^40^ and also to be in strong LD with LCE3C-LCE3B deletion^40^. The third SNP (rs7516108), near the LCE3E gene was independent of LCE3C-3B deletion, showed significant association in the GWAS, however, failed to show any association in the follow up study among Chinese population^18^. All SNPs were genotyped on a 7900HT Fast Real-Time PCR System Instrument using allele-specific Taqman MGB probes labelled with fluorescent dyes FAM and VIC (Applied Biosystems), following manufacturer’s protocols. Allelic discrimination was made with the ABI PRISM 7900HT SDS and the SDS 2.2.2 program (Applied Biosystems). Characteristics of the SNPs studied and their HWE status in our sample are presented in Table S3. Approximately 5% of the samples were randomly selected for Sanger sequencing to check for genotyping errors. More than 99% concordance was observed between these two methods.

### Detection of LCE3C-3B deletion

The status of a 32.2 kb deletion at the EDC comprising LCE3C-3B genes was determined using PCR typing. DNA isolated from blood of healthy individuals and patients were amplified with primer pairs designed to detect normal and deleted (LCE3C_LCE3B-del) alleles. To determine the copy numbers of the deleted allele, we used three-primer assay as described before^40^ with the primer sequences LCE3C-3B_del F: 5′-AATTTTGTGCTTCTGAAATCCA-3′, LCE3C-3B_del_R: 5′-ATTTCATTGAGCAGTGGTTTGT-3′; LCE3B_del_F: 5′- CATTAGCCTGGAGCTTTTGC-3′, LCE3B_del_R: 5′-ACAAGTGATAACATTGTCAGGAGG-3′; LCE3C_del_F: 5′-TTTGGAGCATGTTGTCAGGA-3′, LCE3C_del_R: 5′-AGGGTTAGGCACAGGGAACT-3′. The normal allele could not be amplified owing to its 32.2 kb length, whereas the deleted allele gave around 1088 bps product. The PCR products were then run on 1% agarose gel. With each set of test samples, we used three control DNA (Homozygous non-deleted, homozygous deleted and heterozygous), which were verified by sequencing and blind fold analysis, to check for the PCR failure. To estimate the error rate in genotyping, ~10% of the samples were randomly selected and their deletion status were determined using Taqman qPCR. Three taqman probe-primers (LCE3A Hs00820288_s1, LCE3B Hs04193180_s1, LCE3C Hs00708773_s1; Applied Biosystems) were used to determine the copy number variations in ~10% of the samples along with two known positive controls having homozygous deleted and non-deleted alleles, and a negative control (no template). Each samples were normalized to LCE3A to obtain a ΔCt [Ct of (LCE3B or LCE3C) - Ct of (LCE3A)] for both LCE3B and LCE3C. All samples were then normalized to the positive control to determine ΔΔCt. Copy number variation were calculated by multiplying the relative quantity by 2 [CNV = 2 × 2^−ΔΔCt^]. Average of LCE3B and LCE3C were taken to minimize errors. We found ~98.6% concordance rate between these two independent experiments.

### Gene expression study

Biopsies were snap frozen in liquid nitrogen and grinded using mortar and pestle. RNA extraction was performed using AllPrep DNA/RNA Mini Kit (Qiagen) following the manufacturer’s protocol. Quality of the eluted RNA was checked in Nanodrop Spectrophotometer. One μg of total RNA was used for cDNA synthesis using RevertAid First Strand cDNA Synthesis Kit (Thermo Scientific). The product was subsequently diluted and around 10ng was finally used for each reaction. Transcripts were quantified using a 7900HT Fast Real-Time PCR system (Applied Biosystems) using Taqman probe-primers sets purchased from Applied Biosystems (LCE3A Hs00820288_s1, LCE3B Hs04193180_s1, LCE3C Hs00708773_s1, LCE3D Hs00754375_s1, LCE3E Hs01631234_sH and GAPDH Hs02758991_g1). All values were normalized to the expression of the housekeeping gene GAPDH.

### Statistical Analysis

Case–control analysis was performed to test genetic markers for susceptibility to psoriasis. Hardy–Weinberg equilibrium was evaluated for each SNP using the χ^2^-test. Association for genotype and allele frequencies between cases and controls were calculated using Pearson’s χ^2^-test. Binary logistic regression analysis was carried out using SPSS software package. Haplotype analysis and LD pattern were studied using the SHEsis online software platform^53^. Expression values were compared using unpaired t-test assuming unequal variance. Since the distribution of age and sex were different in cases and controls, we had also checked the association of SNPs after adjusting for age and sex using SPSS. Significant P-values were corrected for multiple testing using Benjamini-Hochberg multiple testing correction in R.

## Additional Information

**How to cite this article**: Chandra, A. *et al.* Increased Risk of Psoriasis due to combined effect of HLA-Cw6 and LCE3 risk alleles in Indian population. *Sci. Rep.* **6**, 24059; doi: 10.1038/srep24059 (2016).

## Supplementary Material

Supplementary Information

## Figures and Tables

**Table 1:** Allele frequencies of the SNPs near LCE3 and CNV of LCE3C-3B.

SNP/CNV	Gene	Alleles	MAF		P-value	Adjusted p-value	OR (95% CI)
**Major/Minor Case Control**							
rs1886734	LCE3A	C/A	0.34	0.37	0.0578	0.0578	1.16 (0.99–1.35)
Deletion	**LCE3C-3B**	**Del/Ins**	**0.35**	**0.41**	0.0018	0.0070	**1.27 (1.09–1.48)**
rs4112788	LCE3D	G/A	0.34	0.37	0.0578	0.0578	1.16 (0.99–1.35)
rs7516108	LCE3E	C/T	0.38	0.42	0.0269	0.0537	1.19 (1.02–1.38)

**Table 2:** Combined effect of HLA-Cw6 with LCE3 risk and non-risk genotypes.

HLA-Cw6							
SNP/CNV	Genotype	ABSENT			PRESENT		
		p-value	OR	95% CI	p-value	OR	95% CI
LCE3A (rs1886734)	AA		1	(reference)	0.0242	2.27	1.10–4.68
	CC/AC	0.9230	0.98	0.68–1.42	**<2.22 × 10^−16^**	**5.32**	**3.52–8.03**
LCE3C-3B Deletion	Ins		1	(reference)	0.0036	2.65	1.36–5.16
	Del/Del-Ins	0.8551	1.03	0.73–1.46	**<2.2 × 10^−16^**	**5.66**	**3.81–8.42**
LCE3D (rs4112788)	AA		1	(reference)	0.0242	2.27	1.10–4.68
	GG/AG	0.9097	0.98	0.68–1.41	**<2.22 × 10^−16^**	**5.32**	**3.52–8.03**
LCE3E (rs7516108)	TT		1	(reference)	4.28 × 10^−05^	3.48	1.88–6.41
	CC/TC	0.2183	1.25	0.88–1.78	**<2.2 × 10^−16^**	**6.73**	**4.49–10.08**

**Table 3:** Stratification based on HLA-Cw6 showed significant association of LCE3 SNPs and LCE3C-3B deletion only in presence of HLA-Cw6.

HLA-Cw6 Present					
SNP	MAF		p-value	OR	95% CI
	Case (N = 326)	Control (N = 106)			
LCE3A	0.30	0.46	4.96 × 10^−05^	1.92	1.40–2.64
DELETION	0.31	0.49	2.09 × 10^−05^	1.98	1.44–2.73
LCE3D	0.30	0.46	4.01 × 10^−05^	1.93	1.41–2.66
LCE3E	0.36	0.48	1.50 × 10^−03^	1.67	1.21–2.29
**HLA-Cw6 Absent**					
	**MAF**		**p-value**	**OR**	**95% CI**
SNP	**Case (N = 376)**	**Control (N = 604)**			
LCE3A	0.37	0.36	0.4863	0.94	0.77–1.13
DELETION	0.38	0.38	0.9806	1.00	0.83–1.20
LCE3D	0.37	0.35	0.4282	0.93	0.76–1.12
LCE3E	0.40	0.40	0.6802	1.04	0.86–1.25
