# Supplementary material for: Increased Risk of Psoriasis due to combined effect of HLA-Cw6 and LCE3 risk alleles in Indian population
Source: Sci Rep. 2016 Apr 6;6:24059. doi: 10.1038/srep24059 (PMC4822143; doi:10.1038/srep24059)

Supplementary Information

Increased Risk of Psoriasis due to combined effect of HLA-Cw6 and LCE3 risk alleles in Indian population

Aditi Chandra[1], *Anirudhya Lahiri*[1], *Swapan Senapati*[2], *Baidehi Basu*[1], *Saurabh Ghosh*[1], *Indranil Mukhopadhyay*[1], *Akhilesh Behra*[3], *Somenath Sarkar*[4], *Gobinda Chatterjee*[3], *Raghunath Chatterjee*[1*]

[1] Human Genetics Unit, Indian Statistical Institute, 203 B. T. Road, Kolkata, India 700108

[2] Consultant Dermatologist, Uttarpara, Hooghly India 712258

[3]Department of Dermatology, SSKM Hospital, Kolkata, India

[4]Department of Dermatology, School of Tropical medicine, Kolkata, India

*Author for correspondence:

E-mail:- rchatterjee@isical.ac.in; Tel:- +91 33 2575 3243; Fax:- +91 33 2577 3049

Supplementary Tables

Table S1. Sample Summary

Sample	Population	Sample Size	Mean Age of Onset/Age (SD)	Male/Female(%)	Type1/Type2(%)
**Case**	**Eastern India**	705	34.96 (15.68) Range : 08-79	68/32	61.74/38.26
**Control**		738	39.24 (14.72) Range : 11-83	52.79/47.21	
**Total**		**1443**	**37.095**	**60.4/39.6**	

Table S2. Association of HLA-Cw6 allele with TypeI and TypeII Psoriasis

	Psoriasis		Normal
	**TypeI**	**TypeII**	
**HLA-Cw6 Frequency**	0.534247	0.34211	0.148501
**P-Value**	**$< 2.2 \times 10^{-16}$**	**1.38×10^{-11}**	
**95% CI**	5.01 - 8.71	2.15 - 4.13	
**Odds Ratio**	6.61	2.98	

Table S3. Characteristics of the SNPs and CNV studied here

SNP ID/CNV	Chromosome	Alleles	Nearest Gene	Position from Gene	HWE (P-values)		
		**Major/Minor**			**All**	**Case**	**Control**
**rs1886734**	1	C/A	LCE3A	3' down (+4168)	0.8881	0.4981	0.443
**Deletion**	1	del/ins	LCE3C-3B	32.2kbp	0.0659	0.6017	0.064
**rs4112788**	1	G/A	LCE3D	3' down (+584bp)	0.7246	0.5593	0.3329
**rs7516108**	1	C/T	LCE3E	5' up (-2981bp)	0.3177	0.6526	0.0847

Table S4. LCE3 SNPs showed similar association level even after adjusting for age and sex

SNP/CNV	Nearest Gene	Genotypes	Genotype Frequency		p-value
		**Major/Hetero/Minor**	**Case**	**Control**	
rs1886734	LCE3A	CC/CA/AA	0.43/0.46/0.11	0.40/0.46/0.15	1.04×10^{-1}
**Deletion**	**LCE 3C-3B**	**Del-Del/Del-Ins/Ins-Ins**	**0.43/0.45/0.13**	**0.37/0.45/0.18**	**3.18×10^{-2}**
rs4112788	LCE3D	GG/GA/AA	0.43/0.46/0.11	0.40/0.45/0.15	1.02×10^{-1}
rs7516108	LCE3E	CC/CT/TT	0.38/0.48/0.14	0.36/0.46/0.19	7.92×10^{-2}

Table S5. Linkage pattern (with r^2 values) of LCE3 cluster

r2: Total	DELETION	rs4112788	rs7516108
**rs1886734**	0.806	0.971	0.610
**DELETION**	-	0.813	0.524
**rs4112788**	-	-	0.613

Table S6: TypeI (Age of Onset<=40 years) showed higher LCE3 association

SNP ID	Control	TypeI (n=438)				TypeII (n=268)			
	MAF	MAF	OR	95% CI	p-value	MAF	OR	95% CI	p-value
**rs1886734**	0.37	0.33	1.20	1.01- 1.43	**0.03958**	0.36	1.01	0.88-1.33	0.4605
**Deletion**	0.41	0.34	1.34	1.12- 1.60	**0.001075**	0.37	1.15	0.94-1.41	0.1694
**rs4112788**	0.37	0.33	1.21	1.02- 1.44	**0.03229**	0.36	1.07	0.87-1.31	0.5352
**rs7516108**	0.42	0.37	1.21	1.01- 1.43	**0.03392**	0.39	1.11	0.90-1.36	0.3187

Table S7: Association model of LCE3E SNP and LCE3C-3B Deletion

		Major(1)	Minor(2)	Case(11/12/22)	Control(11/12/22)	p-value
**rs7516108 (LCE3E)**	Additive	C	T	269/335/97	257/329/137	0.0329
	**Dominant**			**604/97**	**586/137**	**0.00987**
	Recessive			269/432	257/466	0.2692
**LCE3C-3B Deletion**	Additive	Del	Ins	300/313/89	270/329/133	0.0064
	**Dominant**			**613/89**	**599/133**	**0.004069**
	Recessive			300/402	270/462	0.0237

Table S8: Association of SNPs after age and sex adjustment on stratified samples

HLA-Cw6 Present			
**SNP**	**MAF**		
	**Case**	**Control**	**Adjusted p-value**
**LCE3A**	**0.30**	**0.46**	**2.85 x 10^{-4}**
**DELETION**	**0.32**	**0.49**	**2.85 x 10^{-4}**
**LCE3D**	**0.30**	**0.46**	**2.85 x 10^{-4}**
**LCE3E**	**0.36**	**0.48**	**4.20 x 10^{-3}**
**HLA-Cw6 Absent**			
**SNP**	**MAF**		
	**Case**	**Control**	**Adjusted p-value**
LCE3A	0.37	0.36	8.41 x 10^{-1}
DELETION	0.38	0.39	8.41 x 10^{-1}
LCE3D	0.37	0.35	8.41 x 10^{-1}
LCE3E	0.4	0.4	8.41 x 10^{-1}

Supplementary Figures

FigureS1. LD pattern (D') of LCE3 cluster. Total LCE3 cluster showed strong linkage in Indian population.

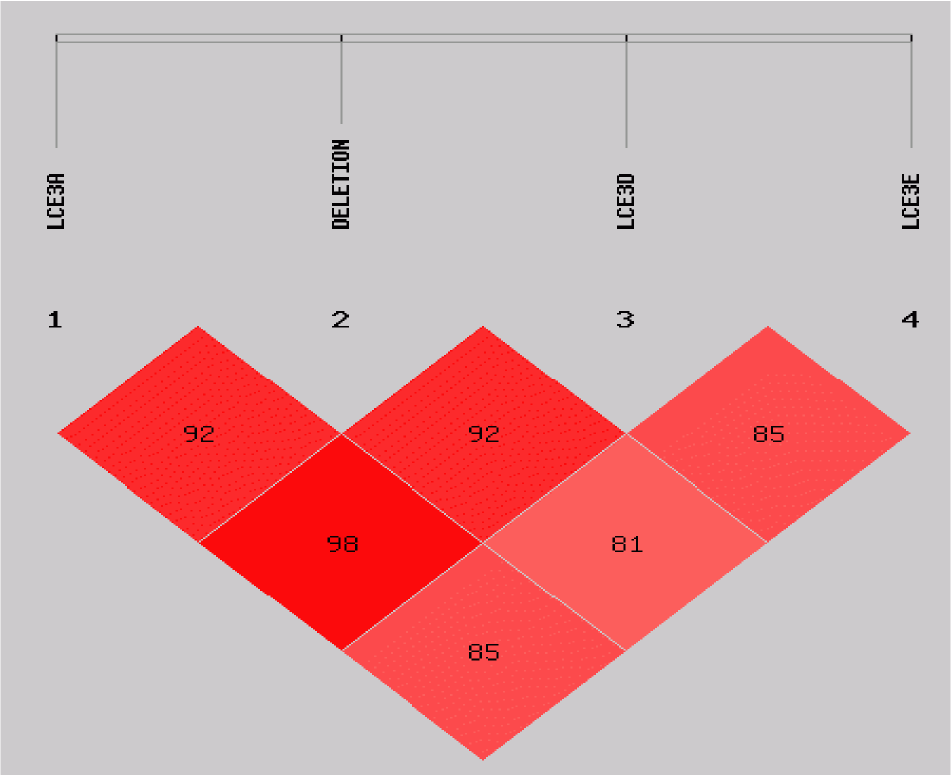

FigureS1b. Variation in expression pattern from diseased tissue with respect to genotypes of LCE3E gene.

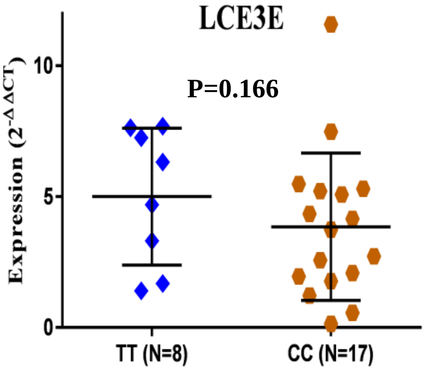

FigureS1c. Expression pattern of LCE3D and LCE3E genes with respect to genotype and HLA-Cw6 status.

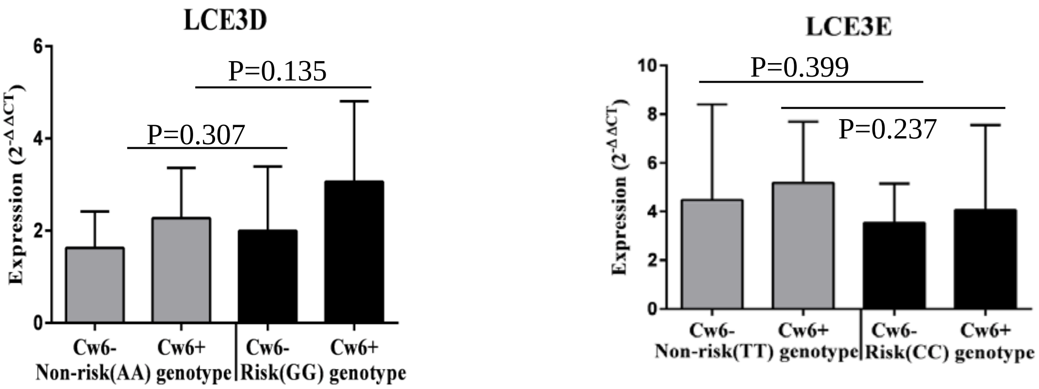

Supplement: Supplementary Information [file srep24059-s1.pdf]
